# Modifiable risk factors for delirium in critically ill trauma patients: a multicenter prospective study

**DOI:** 10.1186/cc14558

**Published:** 2015-03-16

**Authors:** MA Duceppe, A Elliott, M Para, MC Poirier, M Delisle, AJ Frenette, D Deckelbaum, T Razek, M Desjardins, JC Bertrand, F Bernard, P Rico, L Burry, D Williamson, MM Perreault

**Affiliations:** 1 The Montreal General Hospital, Montreal, QC, Canada; 2 Hôpital du Sacré-Coeur de Montreal, QC, Canada; 3 Mount Sinai Hospital, Toronto, ON, Canada

## Introduction

Delirium is associated with significant morbidity and mortality in critically ill medical and surgical patients. However, patients suffering from trauma are generally excluded from these studies. Our objectives were to assess the incidence of delirium and identify modifiable risk factors associated with delirium among critically ill trauma patients.

## Methods

This was a prospective observational study of trauma patients from two critical care trauma centers. We excluded patients who had ICU stay <48 hours and those with severe traumatic brain injury (TBI) (GCS ≤8). Patients were followed until ICU discharge, resolution of delirium, death or ICU length of stay >28 days. Delirium was assessed daily using the Confusion Assessment Method for the ICU until the end of the follow-up period. Demographic and admission data, daily consumption of medications, and environmental factors (that is, presence of clock, TV/radio, and so forth) were collected daily. Univariate analysis was performed using Cox regression analysis to identify risk factors for delirium. The independent effect of modifiable risk factors was assessed using multivariate Cox regression analysis adjusting for severity of illness and nonmodifiable risk factors.

## Results

We enrolled 150 trauma patients resulting mostly from falls (40%) and motor vehicle accidents (28.7%) over 14 months. Patients with TBI accounted for 56.7% while polytrauma patients without TBI accounted for 43.3%. Mean ICU length of stay was 8.1 ± 7.1 days, 69.3% required mechanical ventilation, 14.7% required a tracheostomy. Delirium developed in 58 patients (38.7%) (mean age 62.9 ± 15.7, mean APACHE score 15.4 ± 6.1, mean ISS score 23.4 ± 9.1). Univariate analysis revealed that delirium was significantly associated with the following nonmodifiable risk factors: age (per 10-year range), APACHE II score (per 10-point increase), need of mechanical ventilation, presence of TBI and pre-existing diabetes. In a multivariate analysis when adjusting for the nonmodifiable risk factors, opioids (adjusted HR = 0.37, 95% CI (0.14 to 1.0)), presence of a TV/radio in the room (adjusted HR = 0.28, 95% CI (0.12 to 0.67)), and number of hours mobilized (adjusted HR = 0.77, 95% CI (0.68 to 0.88) had a protective effect on delirium; whereas use of physical restraints (adjusted HR = 2.20, 95% CI (1.11 to 4.35) and active infection (adjusted HR = 2.08, 95% CI (1.16 to 3.71)) remained strongly associated with delirium.

## Conclusion

Considering the long-term consequences of delirium, steps should be implemented to prevent its development in trauma and include optimizing opioids and mobilizing patients while limiting use of physical restraints.

